# *Caenorhabditis elegans* as a Model Host to Monitor the *Candida* Infection Processes

**DOI:** 10.3390/jof4040123

**Published:** 2018-11-07

**Authors:** Asmaa B. Elkabti, Luca Issi, Reeta P. Rao

**Affiliations:** Worcester Polytechnic Institute, Worcester, MA 01609, USA; aelkabti@wpi.edu (A.B.E.); Issiluca@gmail.com (L.I.)

**Keywords:** *Candida albicans*, virulence factors, innate immunity, infectious disease, host-pathogen interactions, model host

## Abstract

*C. elegans* has several advantages as an experimental host for the study of infectious diseases. Worms are easily maintained and propagated on bacterial lawns. The worms can be frozen for long term storage and still maintain viability years later. Their short generation time and large brood size of thousands of worms grown on a single petri dish, makes it relatively easy to maintain at a low cost. The typical wild type adult worm grows to approximately 1.5 mm in length and are transparent, allowing for the identification of several internal organs using an affordable dissecting microscope. A large collection of loss of function mutant strains are readily available from the *C. elegans* genetic stock center, making targeted genetic studies in the nematode possible. Here we describe ways in which this facile model host has been used to study *Candida albicans*, an opportunistic fungal pathogen that poses a serious public health threat.

## 1. Introduction

*Caenorhabditis elegans* is a nematode that is naturally found in soil and compost [1] and has been used as a powerful model organism for more than 50 years. In the 1960s, South African biologist Sydney Brenner pioneered the use of *C. elegans* to study neuronal development and gene expression [2,3] and paved the way for a long lineage of “*C. elegans* scientists” that studied various aspects of cell and animal biology in nematodes. This lineage includes Nobel Prize Laureates Craig Mello and Andrew Fire for their RNAi work [4], Robert Horvitz and John Sulston for their work on organ development and apoptosis [5,6,7], and Martin Chalfie for his work on green fluorescent protein [8]. More recently, *C. elegans* have been used to study muscle development and mechanical function [9,10,11], and tumorigenesis and regulation [12]. Although traditionally used to study molecular and developmental biology, researchers in the past 20 years have also begun to use *C. elegans* to investigate the biology of various human bacterial pathogens including *Pseudomonas aeruginosa*, *Staphylococcus aureus*, *Salmonella enterica*, and *Serratia marcescens* [13,14,15,16,17,18,19], as well as fungal pathogens such as *Cryptococcus neoformans* and *Candida albicans* [20,21,22,23,24,25,26,27,28]. Together, this body of work revealed that many of the mechanisms involved in the human-pathogen interaction are conserved in nematodes, while other immunity mechanisms are unique to the nematode [29,30].

Innate immunity in nematodes is multi-tiered with physical barriers, biochemical, and genetic mechanisms to protect against pathogens. For example, the outer cuticle of the *C. elegans* protects its body from most pathogens. This soil dwelling animal is most susceptible to pathogens through ingestion since they are constantly feeding. The pharyngeal grinder of adult nematodes effectively grinds most bacteria such that no intact bacterial cells are found in their intestinal lumen [31]. Despite this, some microbes are able to colonize the *C. elegans* gut with varying success [32]. More recently, the native microbiome of *C. elegans* was studied and various bacterial strains were identified, with some strains exhibiting antifungal defense potential [33].

**Figure 1:**
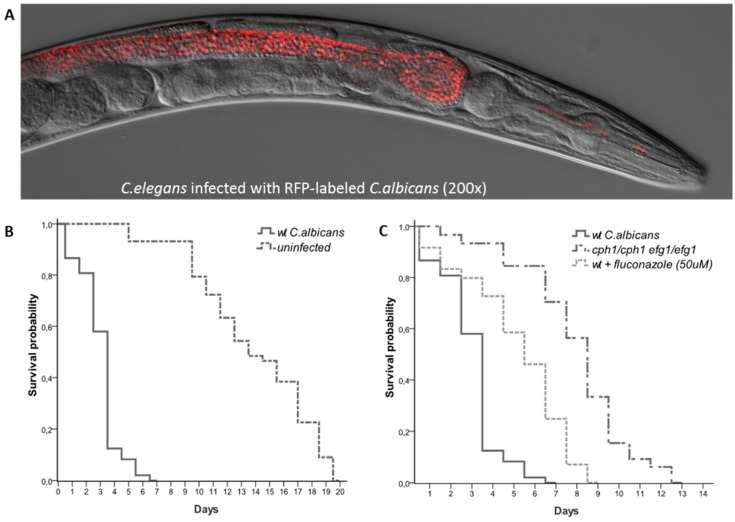
*C. albicans*—*C. elegans* infection model can be pharmacologically or genetically modulated. (**A**) Live cell imaging of *C. elegans* shows *C. albicans* accumulation in the nematode intestinal lumen day 3 post infection. Yeast cells are quickly ingested by the worms and accumulate in the intestinal lumen completely intact indicating that they are able to survive the mechanical crushing of the pharynx. (**B**) Survival curves of nematodes challenged with *C. albicans* versus uninfected controls. (**C**) Survival curves of nematodes challenged with either wild-type *C. albicans*, *cph1/cph1 efg1/efg1* double mutant or wild-type *C. albicans* + 50 mM of fluconazole.

*C. elegans* are either self-fertilizing hermaphrodites or males, have a generation time of 2–4 days, a life span of 2–3 weeks and can be stored frozen almost indefinitely in liquid nitrogen. The nematode is transparent, making it possible to immobilize animals for live cell imaging (Figure 1A). This facilitated the developmental pattern of its 959 somatic cells to be traced through its transparent cuticle. Furthermore, its genome has been sequenced at high coverage and numerous experimental tools are readily available. All of the above qualities, in addition to the availability of functional mutants, fluorescently labeled transgenic strains and RNAi knockdown libraries, make *C. elegans* an extraordinarily powerful model organism.

In its natural habitat, *C. elegans* encounters a variety of threats from ingested pathogens present in the soil and this has provided a strong selective pressure to evolve and maintain a sophisticated innate immune system in its intestinal lumen. Many of the genes and mechanisms involved in the protection of intestinal lumen are orchestrated by highly-conserved elements that also exist in higher mammals [30,34,35]. *C. elegans* therefore represents a great model to study gastrointestinal pathogens such as *Salmonella enterica* [36], *Shigella boydii* [37], *Salmonella typhimurium* [38] or *Vibrio cholera* [39] and fungal species such as *Candida albicans* [26].

Recently our lab and others have shown that *C. elegans* is susceptible to *C. albicans* infections. When *C. albicans* is added to *E.coli* lawns it is ingested by the worms and colonizes the intestine. *C. albicans* infections cause a distinct swelling of the anal region (deformed anal region, Dar) a significantly shorter survival (Figure 1B) and, in certain conditions, *C. albicans* is capable of killing worms by piercing their cuticle [23,24,25,40].

Studies have also shown that many of the genes required for virulence in murine models of infection are also required for virulence in nematodes. For example *RIM101,* a *C. albicans* transcription factor required for alkaline-induced hyphal growth is required for both virulence in murine oropharyngeal candidiasis [41] and virulence in *C. elegans* [23]. Other genes such as *NRG1*, *CAS5*, *ADA2*, *CPH1* and *EFG1* have also shown to be required for virulence in both mice and nematodes [23,28,42] (Figure 1C). These studies clearly demonstrate that many of the conclusions drawn using this simpler organism remain valid in higher mammals, reiterating the utility of *C. elegans* as a model host for the study of infectious diseases. *C. elegans* can also be used for drug discovery (Figure 1C). A high-throughput semi-automated version of the *C. elegans*-*C. albicans* model system was recently used to screen a library of 3,228 compounds [43]. Seven of the 19 compounds capable of extending nematodes survival were known to have antimycotic activity, validating the approach. Taken together these studies show that *C. elegans* can be used as a powerful tool to study, not only the complex host-pathogen dynamics, but also for initial large-scale drug screening [44,45,46,47].

There are an estimated 750,000 hospitalizations and almost 9 million outpatient visits due to fungal infections every year, that amounts to costs of more than $7 billion. These infections can be caused by many *Candida* yeast species, the most common of which is *C. albicans*, and represent the fourth most common cause of healthcare-associated bloodstream infections in the United States [48,49,50,51,52,53]. This polymorphic fungus is part of the human microbiome and it usually lives as a harmless commensal on the skin and mucosa of healthy individuals. However, under certain circumstances, it can cause infections ranging from mild, superficial infections to life-threatening, systemic infections. Even though in the last two decades a large body of literature shed some light on *C. albicans* pathogenicity [54], our understanding of the basic mechanisms that result in this harmless commensal becoming pathogenic remain elusive. In addition, because drug resistance has dramatically increased in the last two decades [55], there is an urgent need to identify new targets to develop better diagnostics and therapeutics. *C. elegans* can be leveraged as the perfect go-between model host between in vitro studies and mammalian models.

## 2. Tools and Techniques

### 2.1. Maintenance of C. albicans and C. elegans

*C. albicans* strains are maintained in YPD (Yeast extract, Peptone, Dextrose) media with or without agar at 30 or 37 °C. Hernday et al. report more detailed methods in *C. albicans* maintenance [56].

Wild type *C. elegans*, N2 (*Caenorhabditis* Genetics Center, U. of Minnesota; https://cgc.umn.edu/strain/N2%20(ancestral)) are typically maintained in nematode growth media (NGM) with or without agar at 20 °C. More detailed methods are described in the WormBook [57]. NGM plates are seeded with *E. coli* OP50, the standard laboratory food for N2 wild type *C. elegans* [2,57]. To test the effects of gene knockdowns, the *E. coli* strain HT115 is used that maintains the RNA interference (RNAi) plasmid [4,10,40,58].

Typically a spot of the food is placed in the middle of the plate and worm eggs or larvae are placed on the pates. Animals freely roam around the plate and feed when they encounter the spot (“small lawn”). *C. albicans* may be mixed in with worm food (OP50). If worms are being tested for their attraction or aversion to a particular (microbial) food they can be exposed to each food by itself. Certain experiments might call for the entire plate to be covered (“big lawn”). This is particularly advantageous to ensure constant exposure to a treatment to identify the mechanism of pathogen resistance—whether it is due to avoidance behavior, or due to innate cellular immunity [59,60].

### 2.2. Measurement of Host Lifespan Post Infection

Overnight cultures of *E. coli* and *C. albicans* are grown overnight in appropriate media and conditions. The cultures are then centrifuged, washed and resuspended to achieve a particular cell density desired for seeding plates. Antibiotics may be used in the feeding mixture depending on experimental objectives and conditions. For example, the food can be a mixture of *E. coli* and *C. albicans* or they can be seeded independently, where the bacteria is used as control and the *C. albicans* as the variable tested. The limitation of the latter is in the age of the synchronized worms used. Since *C. albicans* are too large to be consumed by the larval stage nematode, only L4 and young adult nematodes can be transferred to plates containing *C. albicans* alone, without resulting in starved worms (aka dauer stage, where nematodes under harsh environmental conditions undergo arrest at the second molt [61]). The food is plated in the center of small petri dishes (3.5 cm in diameter) containing NGM agar and incubated overnight at room temperature. The following day, 20 young synchronized adult worms are transferred to the spotted plate (day 0).

Synchronous worm populations are obtained 2–3 days after the addition of *C. elegans* eggs to NGM plates seeded with *E. coli* OP50. In this time frame, the eggs hatch and the larvae reach young adulthood. The worms are scored daily by gentle prodding with a platinum wire and live animals are transferred to new seeded plates grown overnight at room temperature. Daily scoring includes live worms, dead worms and censored worms. Censored animals are those whose death cannot be attributed to the treatment test, like animals accidentally killed while transferring, found dead on the edges of the plates due to drying out, died due to secondary effects of infection, or simply “missing.” *C. elegans* survivals are examined using Kaplan-Meir method and statistical significance evaluated using the log-rank test or Gehan-Breslow-Wilcoxon test, depending on survival trends and fulfilling test assumptions. Additional resources for lifespan assay methods can be found in works by Wilkinson et al. and Amrit et al. [62,63].

### 2.3. Microscopy

The transparent nematodes can be easily visualized and enables live imaging of host-pathogen interactions [64,65]. Typically worms are anesthetisized using sodium azide and placed on an agarose pad containing a drop of M9 buffer. Immobilized worms may be observed under differential interference contrast (Nomarski) and epifluorescence optics. Immunofluorescence can also be used to visualize specific organs or regions of the body [66] for in vivo live visualization. Fluorescently tagged *C. albicans* can be used to infect *C. elegans* to visualize disease progression [25]. As a note of caution, *C. elegans* produce lipofuscin under stress or as they age. This compound fluoresces in blue-green range [67] and may interfere if the tagged proteins are also emitting in the similar range. Therefore, the red/far red fluorophore is effective for visualizing short-term infection [25].

Short-term microscopy of living worms can be conducted using analgesics or anesthetics to immobilize worms and capture images. This method is limited to short-term imaging since worms cannot feed, or will dry out if left for longer periods of time, and then often worms are not recoverable [65]. Alternate methods are necessary for image feeding, locomotion behavioral or physiological responses to stimuli. Different automated approaches have been developed to track the movement and process images of mobile live worms, as reviewed by Husson et al. [68]. Microfluidics is a new approach to handling and capturing images of worms over prolonged periods, and can be used for high throughput assays of RNAi libraries, for instance. Longitudinal observations of individual worms can also be carried out using chambers or droplets. These microfluidics devices are reviewed by San-Miguel and Lu [69]. Recent protocols have also been developed to use multi-channel time-lapse confocal imaging to monitor cell invasion through *C. elegans* basement membranes [70]. Luke et al. also developed some non-microfluidic protocols that enable phase and fluorescent imaging of motile worms [65,71].

## 3. *C. elegans* as a Model Host to Study Virulence of *C. albicans*

### 3.1. Using C. elegans to Study Host Innate Immunity against Fungal Pathogens

The *C. elegans* intestine faithfully mimics mammalian intestines. Like humans, *C. elegans* encounters a variety of threats from ingested pathogens, posing a constant challenge to the innate immune system in its intestinal lumen. Like mammalian intestines, microvilli in *C. elegans* intestine assimilate and digest food while the intestinal luminal cells synthesize and store macromolecules. In addition, the *C. elegans* intestinal microbiota has emerged as a powerful experimental system to study microbial interactions [72,73]. For these reasons, *C. elegans* represents a relevant model to study gastrointestinal pathogens.

**Figure 2:**
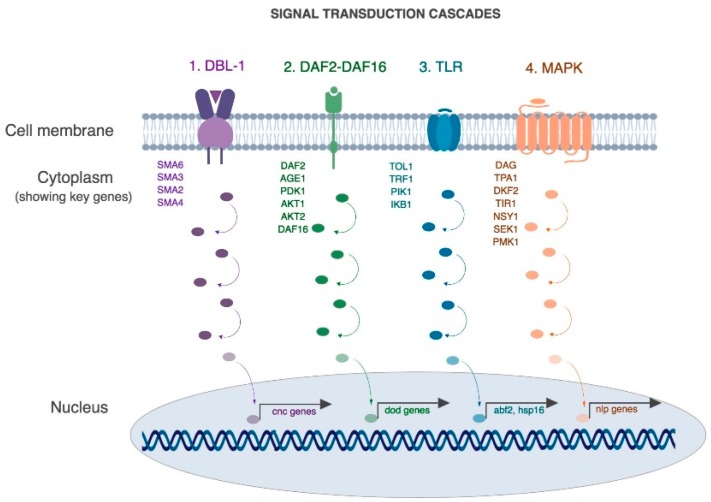
Molecular pathways that play central roles in mounting an immune response in *C. elegans*. (1) TGF-β (or DBL-1) pathway, (2) Insulin signaling pathway, (3) Toll pathway and (4) MAPK pathway. (Collated from [36,74,76,77,78,79,80,81,82,83,84,85]).

A rich body of literature has revealed that many of the mechanisms involved in the human-pathogen interaction are conserved in nematodes [29,30]. For example, four conserved pathways have been shown to be involved in the protection of intestinal lumen in *C. elegans* as well as higher mammals (Figure 2, [30,36,74,75]). A brief description of these pathways as they relate to infection and immunity is described here (Figure 2).

First is the Transforming Growth Factor-β pathway (TGF aka DBL-1 pathway), that regulates the expression of key antimicrobial effectors such as lysozymes and lectins [80]. DBL-1 functions as a dose-dependent ligand that binds heterodimeric receptor DAF-4/SMA-6 triggering a phosphorylation cascade involving SMAD proteins (SMA-2,3,4) and activates SMA-9. SMA-9 controls expression of a key innate immune effector lysozyme (*lys-1* and *lys-8*) [81].

Second, the insulin signaling pathway (aka DAF-2/DAF-16 pathway), is a general stress response pathway that is linked to the expression of multiple antimicrobial proteins such as saponins, thaumatin-like proteins and lysozymes [81,82,83,84]. Briefly, in the absence of microbial threats, the insulin-like peptide DAF-28 binds DAF-2 and triggers a phosphorylation cascade that leads to the cytoplasmic retention of the transcription factor DAF-16. However, in the presence of a microbial threat, the pathway is repressed and DAF-16 phosphorylation triggers expression of antimicrobial peptides.

Third, the Toll pathway (aka TLR pathway) is required for the expression of heat-shock proteins and other defense-like molecules [77] and is implicated in inflammatory microbial interactions [78]. During infection with certain pathogens such as *Serratia mercenses* and *Salmonella enterica* it is required to mount an appropriate avoidance-like immune response [77]. However, in the presence of other pathogens such as *Pseudomonas aeruginosa*, *Microbacterium nematophilum* and *Drechmeria coniospora* this pathway is not required for resistance [79].

Fourth is the mitogen-activated protein kinase (MAPK), which is a well characterized signal transduction cascade in nematodes. It has various biological functions including regulating resistance to bacterial pathogens such as *P. aeruginosa* and *D. consispora* [30,36,74,76]. Briefly, upon activation, DKF-2 activates TIR-1, NSY-1, SEK-1 and PMK-1 in a linear phosphotransfer cascade that leads to the activation of antimicrobial peptides, nlp-29 and nlp-31. The MAPK pathway is a highly conserved pathway and NSY-1, SEK-1 and PMK-1 are the *C. elegans* homologs of human ASK-1, MKK3 and p38 which play major roles in mammalian cellular immune response by controlling the expression of pro-inflammatory cytokines like IL-1 and TNFα.

### 3.2. Other Host Pathways

It is becoming increasingly clear that the resident microbiota plays a significant role in human health and disease [86,87,88,89]. *C. elegans* with its microbial diet is an effective system to study interspecies interactions as well as host-microbe-drug interactions. For example, a systems biology approach was employed to delineate the effects of micronutrients derived from the microbiome on physiology and metabolism of the host. The study connected the flux of micronutrients propionic acid and Vitamin B12 into methionine and folic acid. Interestingly, known orthologs of these genes are present in humans [90,91], suggesting that *C. elegans* is a relevant model for microbiome studies.

### 3.3. Using C. elegans as in vivo Model to Study C. albicans Virulence Mechanisms

**Figure 3:**
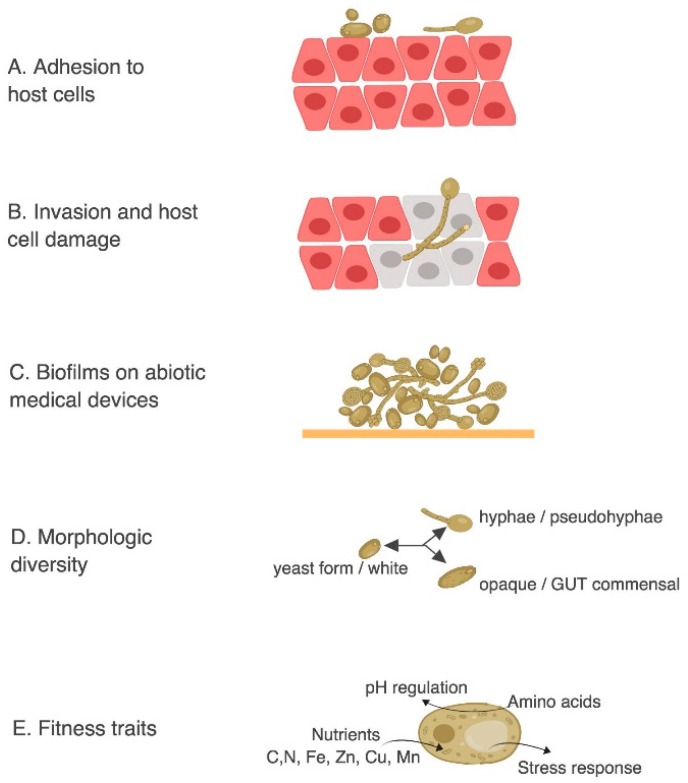
*C. albicans* common virulence mechanisms. Adapted from Mayer et al. [54] (**A**) adhesion to host cells. (**B**) Invasion and host cell damage. (**C**) Biofilms on abiotic medical devices. (**D**) Morphologic diversity. (**E**) Fitness traits: amino acid and nutrient uptake, pH regulation, and stress response.

Powerful reverse genetic approaches have been employed to study *C. albicans* mutants that alter the virulence phenotype in *C. elegans* [23,24]. The most important factor that controls *C. albicans* infection is the physiological status of the host since *C. albicans* infections are commonly found in immunocompromised patients. However, *C. albicans* is also found in approximately 50% of the population as part of the individual’s microflora and it has been shown that slight alterations in the host can turn this normally harmless commensal into a life-threatening pathogen [92]. The transition from harmless commensal to pathogen is a fine line in *C. albicans* and it is attributable to its extensive arsenal of virulence factors. These factors are selectively expressed under suitable predisposing conditions such as the ability to: adhere and penetrate host tissues; form biofilm; switch from yeast-to-hypha; switch from white to opaque state; switch from commensal gut state to virulent state; resist stress, uptake amino acids, adapt to pH fluctuations and utilize extracellular carbon, nitrogen and essential trace metals, just to name a few (Figure 3).

*C. albicans* infections begin with the adhesion of yeast cells to host tissues (Figure 3A). This process is mediated by adhesins proteins such as Als (Agglutinin-Like Sequence) [93]. Als are a family of eight glycosylphosphatidylinositol (GPI)-anchored cell surface proteins that protrude from the *C. albicans* cell wall and that are capable of binding host surface proteins and extracellular matrix [94,95].

Once *C. albicans* attaches to host cells, thigmotropism (contact sensing) [96] triggers *C. albicans* filamentation and allows the pathogen to penetrate deeper host tissues (Figure 3B) via the secretion of hydrolytic enzymes such as Saps (Secreted Aspartyl Proteases) and phospholipases [97,98]. The ability to switch from yeast to hyphae has been shown to be critical for virulence. *C. albicans* can grow in a planktonic yeast form (ovoid shape) that is thought to promote dispersion while hyphal growth of *C. albicans* has an elongated ellipsoid form and can form long filaments that can penetrate deeper tissues (Figure 3B–D). The hyphal form is considered more invasive than the yeast form [99] but the ability to switch from one form to the other is required for virulence. In particular, strains locked in one form or the other have a significantly reduced virulence compared to strains that can freely switch from one form to the other [100]. The hyphal forms are important for tissue invasion in epithelial infections, while yeast form, which are smaller in size and can travel more effectively in the blood stream, are required for systemic infections [101].

*C. albicans* also has a remarkable ability to form biofilms on abiotic surfaces such as catheters, dentures and other implants (Figure 3C). Upon adhesion *C. albicans* proliferates, forms hyphal cells in the upper part of the biofilms, and finally produces a complex extracellular matrix that renders the biofilm more resistant to antimycotic agents [102]. Recent gene expression profiling and ChIP-chip experiments suggest that the cellular circuit controlling biofilm formation is regulated by six transcriptional regulators (*TEC1*, *EFG1*, *BCR1*, *NDT80*, *ROB1* and *BRG1*) that tightly control the expression of ~1000 target genes [103].

Also, *C. albicans* is polymorphic and capable of switching from the an ovoid yeast morphology (white) to distinct elongated cell types that are either mating-competent (opaque^a/α^) or commensal Gastrointestinally-Induced Transition (GUT) forms. These polymorphic forms not only exhibit differences in vitro and in animal models of commensalism and disease but also influence its ability to colonize the host [104]. In particular, white and opaque cells are differentially adapted to specific host niches, with opaque cells being more virulent in the hypoxic environment of the mammalian intestine [105].

In addition to these direct virulence factors, *C. albicans* has various “fitness traits” (Figure 3D) that allow it to grow and resist host immunity. These mechanisms include a robust stress response mediated by heat shock proteins (Hsps) as well as an arsenal of detoxifying enzymes and efflux pumps. Moreover, *C. albicans* has a remarkable ability to adapt to the different nutritional environments of the host. Modulating glycolysis, gluconeogenesis, amino acid uptake and starvation responses are all believed to be important for virulence. For example, in the gastrointestinal tract the concentration of nutrients is relatively high but *C. albicans* competes for nutrients with other members of the microbiome. If the gastrointestinal flora is imbalanced, for example in patients undergoing systemic antibiotics therapies, *C. albicans* has access to additional nutrients and can outgrow the other organisms [106]. On the other hand, when *C. albicans* is in the blood stream, glucose is highly abundant (6–8 mM), but as soon as it is phagocytized, then the nutritional environment changes completely and *C. albicans* faces nutrient starved conditions [107]. Inside the macrophage, *C. albicans* promptly switches from glycolysis to gluconeogenesis and downregulated energetically demanding processes such as ribosome biogenesis [108]. Since the environments that *C. albicans* faces during infection are various, a prompt and efficient metabolic plasticity is required for adapting to such different conditions.

### 3.4. C. elegans as a Live Animal Model for Antifungal Drug Discovery

The increased use of immunosuppressive therapies, potent chemotherapeutic agents and the surge of AIDS patients caused a dramatic increase in the number of immunocompromised patients, which led to a dramatic rise in the number of patients suffering from *C. albicans* infections [109]. In addition to the increased number of *C. albicans* infected patients, the clinical outcome of these infections is complicated by the ability of the strain to develop drug resistance and by the limited number of antimycotics currently available [110,111,112,113]. *C. albicans* developed various clever mechanisms for drug resistance, including genetic alteration of the targeted proteins and overexpression of efflux pumps [114]. Patients infected with drug resistant strains do not respond to the pharmacological therapy and, because the number of antimycotic alternatives is limited, the prognosis can be poor. The large majority of antimycotics currently prescribed have been FDA approved in the 1960s and 1990s and there are not very many drugs in company pipelines that have promising safety and efficacy profiles [115], with the exception of echinocandins such as caspofungin [116,117].

The strength of the nematode model system is that it can be pharmacologically modulated (Figure 1). Fluconazole, amphotericin B and caspofungin [44,118,119,120] alter the course of *C. elegans* infection as measured by extended worm life spans and decreased Dar. Fluconazole and amphotericin B are the most commonly prescribed drugs against *C. albicans* infections, but they have very different mechanisms of action. Fluconazole reduces cell growth by inhibiting the synthesis of ergosterol, a sterol that maintains membrane fluidity. It is considered fungistatic because it can inhibit growth but not cause cell death. On the other hand, amphotericin B is fungicidal because it ultimately kills yeast cells. Amphotericin B directly binds ergosterol, depolarizing the cell membrane, causing leakage of intracellular cations and leading to cell death. A third family of antimycotics was recently discovered: echinocandins, like caspofungin, inhibit 1-3-β-D-glucan synthase [117]—an enzyme that produces 1-3-β-D-glucan, an essential protein in the cell wall of many yeast species including *Candida* [116,121]. Caspofungin has been found to have fungicidal [122,123] or fungistatic effects [124,125] on various fungal species. Fluconazole is more commonly prescribed (can be delivered orally) but poses the threat of generating drug resistance; amphotericin B and caspofungin are more potent but can only be administrated intravenously, where amphotericin B is associated with high nephrotoxicity and caspofungin was associated with relatively low nephrotoxicity and hepatotoxicity [126,127].

The *C. albicans*—*C. elegans* virulence model has been used effectively to screen small molecule libraries combined with a chemical structure analysis to identify novel antifungal agents [43,128,129,130,131,132,133]. Typically, *C. elegans* is infected with *C. albicans* and treated with a library of small molecules. This model host can also be used to test for drug efficacy and toxicity. For example, Filastatin was discovered using *C. elegans* as a live host and has been shown to alleviate disease and improve nematode survival to levels comparable to fluconazole [129] using the *C. elegans* infection model. Furthermore, Filastatin-coated surfaces have been shown to decrease *C. albicans* adhesion [134].

### 3.5. Studying the in vivo Evolution of C. albicans

The nematode virulence model can be used to study the evolution of drug resistance in clinical isolates of *C. albicans*. *C. elegans* has been used as a facile animal model to study the in vivo fitness of several *C. albicans* clinical isolates. Isolates are obtained before and after fluconazole treatment and patients may be sampled multiple times. This serves as a powerful time series to study the progressive evolution of *C. albicans* drug resistance in humans [42].

This study [42] identified single-nucleotide polymorphisms (SNPs), copy number variations (CNV) and loss of heterozygosity (LOH) events that alter *C. albicans* drug resistance and other phenotypes associated with virulence. The ability of each of these strains to form biofilms was determined by testing adhesion to polystyrene, to transition from yeast-to-hyphae and to infect and kill nematodes. These three phenotypes are highly linked to virulence as the ability to form biofilm on medical devices, to transition from yeast-to-hyphae and to kill nematodes, which have been all connected to mammalian virulence [25,135,136].

This study [42] also revealed that LOH events were recurrent and tracked with increased in drug resistance while aneuploidies were transient and did not correlate with fluconazole resistance. The study also identified 240 genes that accumulate persistent SNPs that recur between patients, suggesting that these genes might play a primary role in the complex process of host adaptation and fluconazole exposure. In general, there were substantial variations in the phenotypes observed between isolates. However, *C. albicans in vitro* fitness was shown to be anti-correlated with phenotypic markers of virulence. Furthermore, a progressive increase in drug resistance in vitro was mirrored by a progressive increase in virulence supporting the notion that *C. albicans* adaption during antimycotic therapy relies on its genotypic and phenotypic plasticity [42].

A contributor to this plasticity was recently discovered through the presence of viable haploid and polyploid *Candida* cells in culture. *Candida* was long thought to be an “obligate diploid” but was recently shown to also exist in a viable state as haploid where it can form true hyphae, pseudohayphae and chlamydospores, as well as switch between white-opaque forms [137]. Though haploids grow at slower rates than heterozygous diploids, the haploid cells enabled mating and the production of more adaptable, viable heterozygous diploids. *Candida* in its parasexual phase can develop multi-ploidy which potentiates genetic variation and drives population diversity, increasing probability of adaptation and selection [138] and leading to its remarkable plasticity.

More recently, the *C. elegans* model has been leveraged to study host fitness associated with fungal infection. This study [139] demonstrates that exposure to *C. albicans* as well as other non-*albicans Candida* species affects fecundity, specifically, delayed reproduction, early mortality, reduced brood sizes and delayed reproduction relative to infected healthy hosts.

## 4. Conclusions

*C. elegans* is a good host model organism to study innate immune responses to *Candida* pathogenesis. Its response to infection can often be easily visualized under a dissecting microscope or through immunofluorescence tagging. It is also used for genetic studies and can be used to better understand mechanistic pathways of host-pathogen interaction. Another advantage to using *C. elegans* is the large array of mutants easily attainable from CGC, where high throughput assays can be used for drug screens and to identify pathways of interest. Naturally, there are some limitations to using *C. elegans* as a model host organism since it does not have adaptive immunity and its innate immunity may be less complex than higher organisms. Also, visualization techniques are continuously developing to enhance capturing internal processes in live animals, tracking of motile animals, and automation of high throughput processes.
